# Agreement and differential use of laboratory methods for the detection and quantification of SARS-CoV-2 in experimentally infected animals

**DOI:** 10.3389/fmicb.2022.1016201

**Published:** 2022-11-15

**Authors:** Carla Usai, Lola Pailler-García, Cristina Lorca-Oró, Leira Fernández-Bastit, Núria Roca, Marco Brustolin, Jordi Rodon, Mónica Pérez, Guillermo Cantero, Jorge Carrillo, Nuria Izquierdo-Useros, Julià Blanco, Bonaventura Clotet, Sebastián Napp, Joaquim Segalés, Júlia Vergara-Alert

**Affiliations:** ^1^Unitat Mixta d'Investigació IRTA-UAB en Sanitat Animal, Centre de Recerca en Sanitat Animal (CReSA), Campus de la Universitat Autònoma de Barcelona (UAB), Bellaterra, Catalonia, Spain; ^2^IRTA Programa de Sanitat Animal, Centre de Recerca en Sanitat Animal (CReSA), Campus de la UAB, Bellaterra, Catalonia, Spain; ^3^IrsiCaixa AIDS Research Institute, Badalona, Spain; ^4^Germans Trias i Pujol Research Institute (IGTP), Badalona, Spain; ^5^CIBERINFEC, ISCIII, Madrid, Spain; ^6^Infectious Diseases and Immunity, Faculty of Medicine, University of Vic-Central University of Catalonia (UVic-UCC), Barcelona, Spain; ^7^Department de Sanitat i Anatomia Animals, Facultat de Veterinària, Campus de la UAB, Bellaterra, Catalonia, Spain

**Keywords:** severe acute respiratory syndrome coronavirus 2, RT-qPCR, virus titration, immunohistochemistry, comparison, agreement, tissues, oropharyngeal swab

## Abstract

Rodents are widely used for the development of COVID-19-like animal models, the virological outcome being determined through several laboratory methods reported in the literature. Our objective was to assess the agreement between methods performed on different sample types from 342 rodents experimentally infected with SARS-CoV-2 (289 golden Syrian hamsters and 53 K18-hACE2 mice). Our results showed moderate agreement between methods detecting active viral replication, and that increasing viral loads determined by either RT-qPCR or infectious viral titration corresponded to increasing immunohistochemical scores. The percentage of agreement between methods decreased over experimental time points, and we observed poor agreement between RT-qPCR results and viral titration from oropharyngeal swabs. In conclusion, RT-qPCR and viral titration on tissue homogenates are the most reliable techniques to determine the presence and replication of SARS-CoV-2 in the early and peak phases of infection, and immunohistochemistry is valuable to evaluate viral distribution patterns in the infected tissues.

## Introduction

Since the identification of the severe acute respiratory syndrome coronavirus 2 (SARS-CoV-2) as the causative agent of Coronavirus disease 19 (COVID-19), there has been a global effort to develop animal models for the preclinical evaluation of vaccines and therapeutic agents. For emerging infectious diseases with a potentially lethal outcome, as for COVID-19, animal models are crucial prior to the development of any medical counteraction, to understand the pathogenesis, the molecular interactions between the infectious agent and the host, and the dynamics of disease progression. The characterisation and validation of such models require the study of viral replication, clinical signs, pathological features, immune response, transmission patterns and the effect of demographic characteristics using different laboratory methods (Muñoz-Fontela et al., 2020, 2022). Often animal species are not naturally susceptible to human pathogens, and the development of humanised or transgenic strains is necessary (Swearengen, 2018).

In February 2020, the World Health Organization established an *ad hoc* Expert Group focused on COVID-19 disease modelling (WHO-COM) to accelerate progress and reduce duplication of effort. Amongst the small animal models, golden Syrian hamsters (GSH), as well as mice expressing the human Angiotensin-Converting Enzyme 2 (hACE2, the main receptor used by the virus to bind and enter cells) under the control of the human cytokeratin 18 promoter (K18-hACE2 mice), have proved particularly relevant, despite presenting some significant differences with the human disease. In particular, the hamster model shows extensive apoptosis in the lungs which is not reported in human pulmonary lesions, whilst the K18-hACE2 mice model is characterised by lethal brain lesions (Chan et al., 2020; Moreau et al., 2020; Vidal et al., 2022).

Here we report a pooled analysis of data produced by our group from a total of 342 animals (including GSH and K18-hACE2 mice) inoculated with the D614G variant, characterised by an aspartic acid to glycine shift at the amino acid position 614 of the Spike protein, and dominant worldwide during the early phase of the pandemic (Korber et al., 2020). The methods considered in the present study are routinely applied for laboratory diagnosis of viral diseases. Quantitative real-time polymerase chain reaction (qPCR) is often considered the gold standard for pathogen detection; several PCR protocols have been validated and widely used during the first 2 years of the SARS-CoV-2 pandemic to confirm positive results of point-of-care tests in patients (Liu et al., 2020; Lieberman et al., 2020; Zhen et al., 2020). Moreover, immunohistochemistry (IHC) relies on specific monoclonal or polyclonal antibodies to determine the presence of a specific microorganism in tissue samples (Rockx et al., 2020; Oumarou Hama et al., 2022). On the other hand, the observation of virus-induced cytopathic effect (CPE) in permissive cell lines is one of the gold standards for titrating, isolating and identifying viruses, and it allows the quantification of infectious viral particles (viral titration, VT). Since SARS-CoV-2 is known to cause CPE characterised by multinucleated syncytia formation and apoptosis both *in vitro* and *in vivo*, this technique, although time-consuming, is a valuable tool for the diagnosis of active infections and the quantification of viral load (Fenner et al., 1987; Buchrieser et al., 2020; Zhu et al., 2020).

This work aimed to determine the degree of concordance between the diagnostic methods applied in previous studies and to identify the most robust tools for the characterisation of animal models of SARS-CoV-2 infection. Here we show a moderate agreement between techniques detecting active viral replication, partly influenced by the time post-infection and the sample type analysed. We also found that increasing viral loads determined by viral titration correlated with IHC scores, confirming the relevance of IHC in the evaluation of viral distribution patterns, and in assessing the association between tissue lesions and viral presence. Since all experimental studies were performed with the same variant, our results are not affected by the variability associated with different variants of concern (VOC).

To our knowledge, this is the first side-to-side comparison of the performance of such methods under experimental settings, and it has the potential to contribute to accelerating the progress in the field as well as suggesting a more efficient allocation of laboratory resources.

## Materials and methods

### Sample selection

This integrated analysis includes data from GSH and K18-hACE2 mice experimentally inoculated with SARS-CoV-2. RT-qPCR and viral titration were performed from oropharyngeal swabs (OS), nasal turbinate (NT), and lung (L) tissues from both species, and brain (B) from mice. Nucleoprotein (NP)-specific IHC was performed on all tissue samples. The samples were generated in eight different studies, six performed in GSH and two in K18-hACE2 mice. All animals (*n* = 342) were intranasally inoculated with 10^3^, 10^4^, or 10^5.8^ TCID50/ml of the hCoV-19/Spain/CT-2020030095/2020 (GISAID ID EPI_ISL_510689) isolate (D614G variant), each animal receiving a total volume of 100 μl (50 μl/nostril). Depending on the study, some animals received treatments such as prophylactic vaccination or post-inoculation antiviral treatments. Samples were collected 2-, 4-, and 6 or 7-days post-inoculation (dpi) as a final point. Table 1 summarises the distribution of sex and treatment status of the animals from which samples were collected at each time point. Animal experiments were approved by the Institutional Animal Welfare Committee of the *Institut de Recerca i Tecnologia Agroalimentàries* and by the Ethical Commission of Animal Experimentation of the Autonomous Government of Catalonia and conducted by certified staff. Experiments with SARS-CoV-2 were performed at the Biosafety Level-3 facilities of the Biocontainment Unit of IRTA-CReSA (Barcelona, Spain).

**Table 1:** Distribution of animals included in the study per sex, time of sacrifice, and treatment status.

	Treatment	2 dpi		4 dpi		6–7 dpi		Total	
		Males	Females	Males	Females	Males	Females	Males	Females
Hamsters	Infection	24	27	25	30	30	33	79	90
	Vaccine candidates 1 dose	9	8	14	15	14	15	37	38
	Vaccine candidates 2 doses	8	7	7	8	8	7	23	22
	Total *N* = 289	**41**	**42**	**46**	**53**	**52**	**55**	**139**	**150**
Mice	Infection	3	3	8	4	9	10	20	17
	Antiviral	0	0	0	8	0	8	0	16
	Total *N* = 53	**3**	**3**	**8**	**12**	**9**	**18**	**20**	**33**
Total *N* = 342								**159**	**183**

dpi, day post inoculation.

### Laboratory detection of SARS-CoV-2

RT-qPCR, IHC and viral titration procedures are described elsewhere (Brustolin et al., 2021; Vidal et al., 2022). Briefly, RT-qPCR was performed to detect viral genomic RNA (gRNA) and subgenomic RNA (sgRNA) in two separate reactions, using in both cases Envelope (E)-specific primers and probes targeting regions upstream of the E gene (UpE; Corman et al., 2020; Wölfel et al., 2020). IHC was performed on formalin-fixed tissue slides using a Nucleoprotein (NP)-specific monoclonal primary antibody (Sino Biological, ref. 40143-R019; Buchrieser et al., 2020; Zhu et al., 2020). Viral titration by CPE assay was performed *in vitro* on Vero-E6 cells (ATCC CRL-1587) and the median tissue culture infectious dose (TCID_50_/ml) was calculated with the Reed-Muench method (On the Calculation of TCID50 for Quantitation of Virus Infectivity | SpringerLink, 2022).

### Definition of variables and interpretation of the results

The results were expressed as cycle threshold (Ct) values for both gRNA and sgRNA RT-qPCR, TCID_50_/ml for the CPE assay, and a semi-quantitative score ranging from 0 to 3 for IHC. Samples with undetermined Ct values or Ct > 36.8 were considered RT-qPCR negative. Samples below the limit of quantification (LOQ, <10^1.8^ TCID_50_/ml) of the CPE assay were considered negative, as well as those with no CPE in any of the wells. Increasing IHC scores indicate increasing amounts of detectable viral antigen (0: absence of viral antigen; 1: low amount, multifocal localisation; 2: moderate amount, multifocal localisation; 3: high amount, diffuse localisation); score assignment was performed in blind.

### Comparison between different technique results

All statistical analyses were performed using R programming language (version 4.2.0, R Core Team, 2022) and the RStudio environment (RStudio Team, 2022), with stats, DescTools, epiR, ggplot2, and tidyverse packages.

### Dichotomous comparison

The numerical results were converted into dichotomous variables (positive/negative) following the criteria described above. To determine the agreement between the dichotomised results of different methods, contingency tables were created, and Cohen’s kappa coefficient was computed considering all possible pairwise comparisons (Watson and Petrie, 2010; Kwiecien et al., 2011).

### Assessment of the correlation between positive results

In order to evaluate the association between the diagnostic test’s measures on a continuous scale (i.e., gRNA RT-qPCR, sgRNA RT-PqPCR and VT), the correlation between tests was assessed. This allows us to measure the strength and direction of the association between the positive results of those methods. Since gRNA RT-qPCR, sgRNA RT-qPCR and VT are measured on different scales, the results had to be standardised to be comparable, for which the mean was substracted and the result was divided by the standard deviation. The Spearman’s correlation test was then performed, because of the lack of normality of the data. Also, as recommended by Watson and Petrie (2010) the Lin’s concordance correlation coefficient (CCC) was calculated.

### Distribution of positive results according to their IHC score

The distribution of standardised positive results of RT-qPCR and VT was compared across positive IHC semi-quantitative scores. Kruskal–Wallis test and Dunn’s test for multiple comparisons with Bonferroni correction were performed.

### Relationship of experimental results with other covariates

#### Dichotomous comparison

The numerical results were converted into dichotomous variables (positive/negative) as described above. For each species, sex, time-point (days post-inoculation), treatment, and tissue type, a Chi-squared test with Bonferroni correction was performed to compare the percentage of agreement between methods (pairwise). This analysis was performed by categorising the results into “Agree” or “Disagree.”

#### Distribution of IHC scores according to each covariate

For each species, sex, time-point, treatment status and tissue type, a Chi-squared test with Bonferroni correction was performed to compare the percentage of samples assigned to each IHC score. The same analysis was performed on the entire dataset and the hamster and mice datasets separately.

## Results

### Composition of the pooled data

Results obtained with each method were dichotomised into positive and negative as shown in Table 2. gRNA RT-qPCR data were available for all samples; other methods were performed only on a fraction of the samples, depending on the objective of each study.

**Table 2:** Frequency of the dichotomised results (positive/negative) by laboratory methods.

	gRNA RT-CR	sgRNA RT-PCR	VT	IHC	Total
Negative	86	304	439	123	**952**
Positive	901	513	456	329	**2,199**
Total	**987**	**817**	**895**	**452**	

RT-qPCR, reverse transcription followed by quantitative polymerase chain reaction; gRNA, genomic RNA; sgRNA, subgenomic RNA; VT, infectious viral titration; IHC, immune histochemistry.

### Moderate agreement between methods detecting actively replicating virus

A moderate concordance was found between methods indicative of active viral replication (sgRNA RT-qPCR, IHC and VT), whilst Cohen’s kappa coefficient was lower between gRNA and the other methods, reaching the “moderate” range only when considered in combination with IHC (Table 3A). Reducing the dataset to the samples that were analysed using all available methods (Table 3B), the concordance between sgRNA and VT was substantial; similarly, the kappa coefficient between gRNA and VT increased, indicating a fair agreement; all other pairwise comparisons maintained the same concordance ranges.

**Table 3:** Qualitative assessment of the techniques used in the experimental studies calculated on (A) the entire dataset or (B) selecting the samples analysed by all methods.

(A)				
Techniques	*N*	Proportion of agreement	Kappa	95% CI
gRNA vs sgRNA	816	71.94%	0.29	[0.24, 0.34]
gRNA vs VT	894	59.17%	0.17	[0.13, 0.21]
gRNA vs IHC	445	83.60%	0.49	[0.40, 0.57]
sgRNA vs VT	739	78.08%	0.56	[0.49, 0.63]
sgRNA vs IHC	440	78.18%	0.52	[0.43, 0.61]
VT vs IHC	424	79.01%	0.52	[0.43, 0.61]
**(B)**				
**Techniques**	***N***	**Proportion of agreement**	**Kappa**	**95% CI**
gRNA vs sgRNA	424	71.46%	0.30	[0.24, 0.37]
gRNA vs VT	424	73.11%	0.32	[0.25, 0.39]
gRNA vs IHC	424	84.20%	0.47	[0.39, 0.56]
sgRNA vs VT	424	86.08%	0.70	[0.61, 0.80]
sgRNA vs IHC	424	78.30%	0.51	[0.42, 0.60]
VT vs IHC	424	79.01%	0.52	[0.43, 0.61]

Cohen’s kappa coefficient was computed; 0 ≤ kappa ≤ 0.2: slight agreement; 0.2 < kappa <=0.4: fair agreement; 0.4 < kappa ≤ 0.6: moderate agreement; 0.6 < kappa ≤ 0.8: substantial agreement; kappa > 0.8 almost perfect agreement. CI, confidence interval; gRNA, genomic RNA; sgRNA, subgenomic RNA; VT, infectious viral titration; IHC, immune histochemistry.

### Significant correlation between standardised results

**Figure 1:**
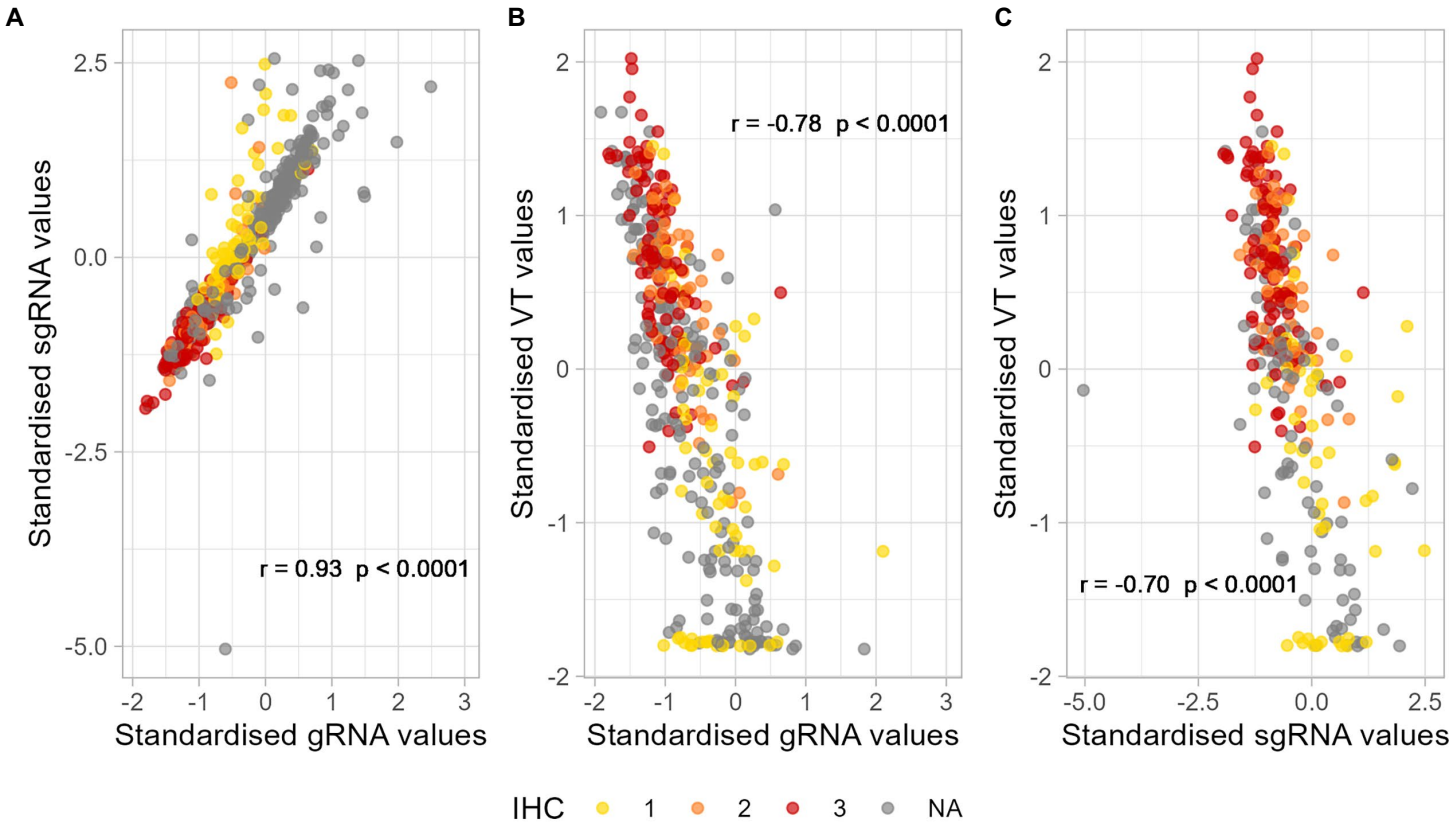
Correlation between the standardised results of positive samples by **(A)** sgRNA and gRNA RT-qPCR, **(B)** VT and gRNA RT-qPCR, and **(C)** VT and sgRNA RT-qPCR. Different colours represent semiquantitative IHC scores assigned to positive samples. *p*-Values lower than 0.05 are considered significant. **(A)** *r* = 0.93, *p*-value < 0.0001. **(B)** *r* = −0.78, *p*-value < 0.0001. **(C)** *r* = −0.70, *p*-value < 0.0001. *r*, Spearman’s correlation coefficient; RT-qPCR, reverse transcription followed by quantitative polymerase chain reaction; gRNA, genomic RNA; sgRNA, subgenomic RNA; IHC, immune histochemistry; VT, infectious viral titration. NA, non-available.

Next, we wanted to determine whether the samples with positive results by the different methods were correlated. Therefore, the concordance correlation coefficient (CCC) for each pair of numerical methods was calculated. Results indicated a positive, highly correlated relationship between gRNA and sgRNA (CCC = 0.76 with 95% CI = [0.73, 0.78]), a negative, moderate correlation between gRNA and Viral titration (CCC = −0.48 with 95% CI = [−0.54, −0.41]), and negative, moderate correlation between sgRNA and Viral titration (CCC = −0.50 with 95% CI = [−0.58, −0.41]). Negative relationships are expected due to the nature of the techniques. Similar results were found calculating the Spearman’s correlation coefficient, where all techniques showed statistically significant correlations; such results seemed independent of the (positive) IHC scores assigned to samples (Figure 1).

### Differences in viral load according to the IHC score

**Figure 2:**
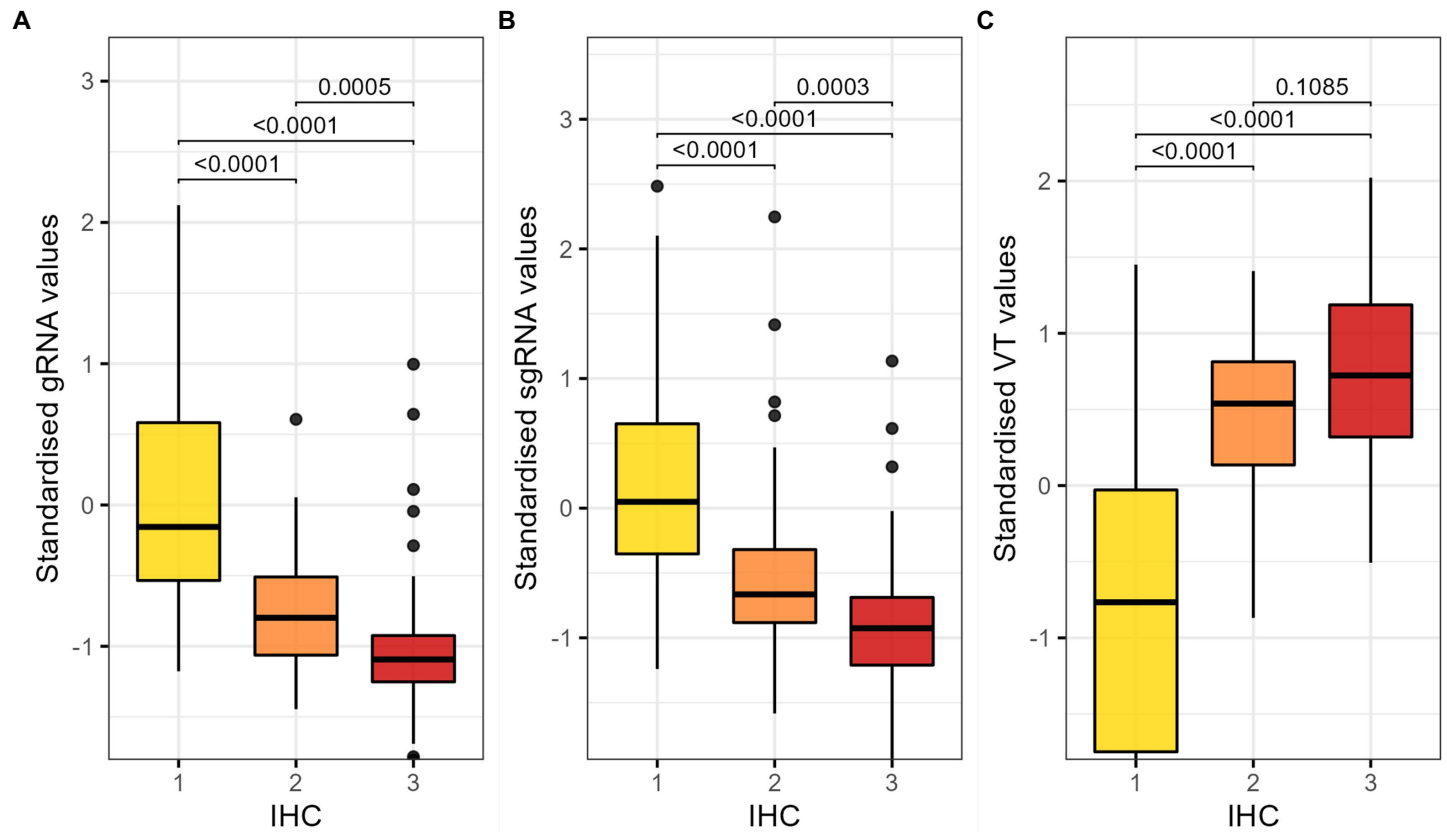
Comparison of the distribution of standardised **(A)** gRNA RT-PCR, **(B)** sgRNA and **(C)** VT results by IHC score. Kruskal-Wallis test and Dunn’s test for multiple comparisons with Bonferroni correction; *p*-values lower than 0.05 are considered significant. RT-qPCR, reverse transcription followed by quantitative polymerase chain reaction; gRNA, genomic RNA; sgRNA, subgenomic RNA; IHC, immune histochemistry; VT, infectious viral titration.

We compared the distribution of standardised RT-qPCR and VT results across the different IHC scores. We found statistically significant differences in gRNA and sgRNA values between samples with different IHC scores, as well as in the viral load determined by VT (Figure 2).

### Influence of other covariates on the agreement between laboratory methods

**Figure 3:**
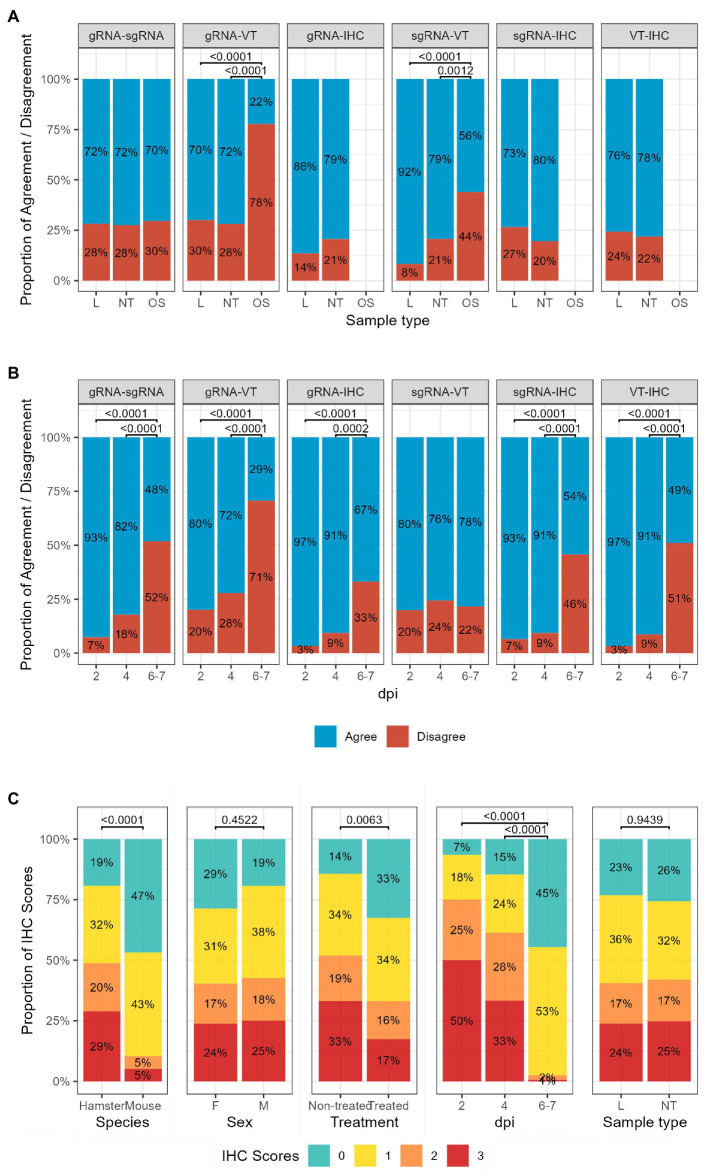
Influence of **(A)** sample type and **(B)** day post-infection on the percentage of agreement between techniques. **(C)** The proportion of IHC scores is different across time points, species, treatment status and sexes. Chi-squared test followed by Bonferroni correction; *p*-values lower than 0.05 are considered significant. L, lung; NT, nasal turbinate; OS, oropharyngeal swab; gRNA, genomic RNA; sgRNA, subgenomic RNA; IHC, immune histochemistry; dpi, day post-inoculation; F, females; M, males.

The results of pairwise comparisons were categorised into “Agree” and “Disagree”, and the proportion of agreement between each pair of techniques was compared across sexes, species, sample types, dpi, and treatment status. No differences were found across sexes, species, or treatment status (Supplementary Table 1). When we considered the sample type as a covariate, the percentage of agreement between the VT and both gRNA and sgRNA results was lower for OS than for other sample types (NT, L; Figure 3A). The comparison of the results obtained at different time points showed that the percentage of agreement decreased over time in all pairwise comparisons, except for sgRNA versus VT. The percentage of agreement between techniques on samples collected at 6–7 dpi was significantly lower than the percentage of agreement between the same methods on samples collected at 2 and 4 dpi (Figure 3B). Also, the proportion of IHC scores at 6–7 dpi was significantly different from the distributions recorded at earlier time points (Figure 3C); a strong increase in the percentage of samples with no or lower amount of viral antigen was observed at the expenses of the proportion of samples with moderate and high amounts. A statistically significant difference in IHC scores was also found between samples from treated and non-treated animals, and between GSH and K18-hACE2 mice (treated animals and K18-hACE2 mice had a higher percentage of negative and slightly positive samples).

### Independent analyses of hamster and mice datasets

The same analyses were performed separately for hamsters and mice (Supplementary Tables 2, 3; Supplementary Figure 1). GSH results (Supplementary Table 2) showed that the proportion of IHC scores by other covariates was consistent with that observed in the entire dataset, with significant differences between 6 and 7 dpi and earlier time points (Supplementary Figure 1A). In the case of results obtained in K18-hACE2-mice (Supplementary Table 3), sgRNA and VT showed a higher Cohen’s kappa coefficient than the entire dataset, indicating substantial agreement. We also found that more covariates influenced the agreement between techniques performed in K18-hACE2 mice; in particular, statistically significant differences between sexes (in the comparison between sgRNA and IHC), time-points (the agreement between gRNA and sgRNA, and sgRNA and VT increased over time, whilst decreased between the VT and IHC) and sample types (B recorded the highest percentage of agreement, OS the lowest) were found. The distribution of IHC scores was significantly different in all comparisons performed (Supplementary Figure 1C).

## Discussion

The development of animal models has been instrumental in the study of SARS-CoV-2 infection and the evaluation of medical interventions. The characterisation and validation of new animal models entail the study of multiple parameters, using a variety of laboratory methods. Here we report the side-by-side comparison of four different analytical methods for the detection of SARS-CoV-2 in experimentally inoculated GSH and K18-hACE2 mice: RT-qPCR detection of gRNA and sgRNA, IHC, and VT by the observation of CPE *in vitro*, performed on OS, NT, L, and B samples collected at 2, 4, and 6–7 dpi.

gRNA RT-qPCR is used to determine the presence of the virus through the detection of its genetic material, without any information on its replicative potential. On the other hand, sgRNA molecules are produced only during the coronavirus replication cycle and have been used as a proxy of infectivity (Wölfel et al., 2020; Bruce et al., 2022). Similarly, IHC detects the presence of viral proteins inside the cells, produced during active infection (Lieberman et al., 2020). We dichotomised the results into positive and negative to determine the agreement between methods by computing Cohen’s kappa coefficient, which gives a quantitative assessment of how well two tests agree by comparing the observed rate of agreement with the rate expected by chance (Watson and Petrie, 2010; Kwiecien et al., 2011). Methods determining active replication showed a higher kappa coefficient between each other than with the detection of gRNA. RT-qPCR and VT results, both indicating viral load either indirectly or directly, respectively, are expressed in different units and scales; therefore, we standardised their numerical results to compare the correlation between the positive results obtained for the same samples with the different methods. We found a statistically significant correlation between the results of all techniques (analysed pairwise). This suggests that RT-qPCR (for both gRNA and sgRNA) and VT have similar abilities to discriminate amongst different levels of positivity. Also, statistically significant differences between groups were found in the distribution of standardised viral loads across the different IHC scores, indicating that, as expected, higher viral loads corresponded to higher amounts of viral proteins in the infected tissues.

We next analysed the effect of covariates on the concordance between techniques and found that species, sex, and treatment status did not influence the results, suggesting that the methods we used were equally reliable irrespective of the mentioned covariates. On the contrary, the percentage of agreement was influenced by the experimental time point and the sample type. The percentage of agreement amongst SARS-CoV-2 detection methods decreased over time for all pairwise comparisons, being significantly lower at 6–7 dpi than at earlier time points; the only exception being the comparison between sgRNA and VT. The disagreement recorded between gRNA and the results indicative of viral replication is reflected by the lower kappa coefficient as previously discussed. In particular, the lower percentage of agreement between sgRNA and VT versus IHC might be due to the low amount and multifocal distribution of antigen at later time points (Supplementary Figure 2). Accordingly, the distribution of IHC scores across time points shows that at 6–7 dpi the proportion of negative IHC samples increases at the expense of the proportion of samples assigned 2 and 3 scores, consistent with ongoing viral clearance. This result suggests that IHC should be preferentially used not as a method to determine viral infection, but rather as a follow-up technique to monitor antigen production and clearance/retention. Additionally, its combination with histology represents a powerful tool to study the association of the viral antigen with inflammation or other pathological features.

Considering the sample type as a covariate, the percentage of agreement between VT and both gRNA and sgRNA obtained in OS was significantly lower than in the tissue homogenates. This difference might be explained by the different nature of the sample, since the OS collects what is released by the tissue, being, therefore, an indirect approximation of the molecular events taking place inside the tissue. OS were performed instead of nasal swabs for technical reasons (nasal cavities in GSH and mice are very small and therefore of difficult access for sampling), but our results suggest that OS are not the most adequate sample type for the diagnosis of SARS-CoV-2 infection in these animal models.

On the other hand, the percentage of agreement between all pairs of methods was not statistically different between L and NT, reflecting that both tissues have a similar capability of supporting replication of the SARS-CoV-2 variant used in the studies.

Interestingly, we observed different effects of covariates when analysing GSH and K18-hACE2 mice separately. K18-hACE2 mice showed a significant effect of sexes and an increase in the percentage of agreement over time for some comparisons. Unlike GSH, K18-hACE2 mice constitutively express the hACE2 receptor on virtually every cell type, therefore allowing viral entry and potential replication in tissues not infected in natural hosts for SARS-CoV-2 infection. It was shown that both SARS-CoV and SARS-CoV-2 cause lethal infection in K18-hACE2 mice, invading the brain and causing acute meningoencephalitis (McCray et al., 2007; Vidal et al., 2022). For these reasons, we included brain tissue samples in this analysis, which showed the highest percentage of agreement between techniques, reflecting a high proportion of infected samples, and usually with moderate to high viral load. We also found that the percentage of agreement between sgRNA and IHC was lower in male mice than in female mice; this might be explained by actual differences in the infection dynamic and lesions between male and female subjects, as already shown in natural infection in humans where a gender-biased risk factor was observed (Dorjee et al., 2020). However, our results might have been skewed by the fact that within our mice subgroup, only females were treated with antivirals: a bigger and more balanced sample is needed for a correct interpretation. The increase in the percentage of agreement over time in K18-hACE2 mice can also be explained by the different natural history of SARS-CoV-2 infection in this host: unlike hamsters, K18-hACE2 mice are not able to clear the virus within a week, therefore there is still a high proportion of positive samples detected 6–7 dpi (Buchrieser et al., 2020). The differences observed between hamsters and mice in our analyses stress the importance of choosing the correct experimental setting for each study. It is crucial, therefore, to have a clear understanding of the differences between the experimental animal model and the human disease, and, as more VOCs are characterised, of the natural history of every specific VOC.

According to the obtained results, gRNA RT-qPCR and VT on tissue homogenates were the most reliable techniques to determine the presence and the replication of SARS-CoV-2, respectively, especially in the early and peak phases of the infection. sgRNA detection by RT-qPCR has been used as a proxy for viral replication, but the results reported so far are controversial. Some authors suggested that the absence of sgRNA could be used as a test to rule-out active infection, but it should not be considered as proof of infectiousness. In fact, others have shown that sgRNA can be detected even after seroconversion when the shedding of the infectious virus has stopped (Wölfel et al., 2020; van Kampen et al., 2021; Bruce et al., 2022). We, therefore, consider that VT is to be preferred to sgRNA RT-qPCR whenever the infectious capacity of the samples at any time point is of interest, since in some cases sgRNA positive samples were not able to induce any CPE on permissive cells. On the other hand, to maximise the sensitivity of SARS-CoV-2 genetic material detection, the use of gRNA RT-qPCR is suggested, since it has proven able to detect the highest number of positive samples in our studies. We also consider the use of IHC as a valuable tool for the evaluation of viral distribution patterns, also in light of the good agreement with both sgRNA RT-qPCR and VT at the beginning and peak of the infection. Despite being used mainly as a qualitative or semi-quantitative assay, IHC has the advantage of being easily performed on different tissue portions at the same time, allowing a more complete study of viral tropism and distribution, which might be not uniform throughout the infected organ. In conclusion, all analytical methods considered in this study allowed the detection of SARS-CoV-2 presence and/or replication in different samples of these two animal models of COVID-19-like disease; nonetheless, the choice of the laboratory method must be accurately guided case by case, driven by the objective of each study.

## Data availability statement

The original contributions presented in the study are included in the article/Supplementary material, further inquiries can be directed to the corresponding author.

## Ethics statement

The animal study was reviewed and approved by IRTA and Generalitat de Catalunya.

## Author contributions

CU, LP-G, JS, and JV-A conceived and designed the experiments. CL-O, LF-B, MB, GC, JS, and JV-A performed the animal procedures. CU, LP-G, CL-O, LF-B, NR, MB, JR, MP, JS, and JV-A performed the analytical experiments. CU, LP-G, JC, NI-U, JB, BC, SN, JS, and JV-A analysed and interpreted the data. CU, LP-G, JS, and JV-A wrote the paper. All authors contributed to the article and approved the submitted version.

## Funding

The research of the CBIG consortium (constituted by IRTA-CReSA, BSC & IrsiCaixa) is supported by Grifols. This work was also supported by CERCA Programme/Generalitat de Catalunya, and the crowdfunding initiative #joemcorono (https://www.yomecorono.com). NI-U is supported by the Spanish Ministry of Science and Innovation (grant no. PID2020-117145RB-I00), EU HORIZON-HLTH-2021-CORONA-01 (grant no. 101046118) and by institutional funding from Grifols, Pharma Mar, HIPRA, Amassence and Palobiofarma. The funders had no role in the design of this study and did not have any role during its execution, analyses, interpretation of the data, or decision to submit results.

## Conflict of interest

The authors declare that the research was conducted in the absence of any commercial or financial relationships that could be construed as a potential conflict of interest.

## Publisher’s note

All claims expressed in this article are solely those of the authors and do not necessarily represent those of their affiliated organizations, or those of the publisher, the editors and the reviewers. Any product that may be evaluated in this article, or claim that may be made by its manufacturer, is not guaranteed or endorsed by the publisher.
