# Supplementary material for: Agreement and differential use of laboratory methods for the detection and quantification of SARS-CoV-2 in experimentally infected animals
Source: Front Microbiol. 2022 Nov 15;13:1016201. doi: 10.3389/fmicb.2022.1016201 (PMC9706237; doi:10.3389/fmicb.2022.1016201)
Supplement: Supplementary file 1 [file Data_Sheet_1.docx]

Supplementary Material

**Supplementary Table 1. No differences between sex, species, or treatment status; analysis performed on the entire dataset.** Chi-squared test followed by Bonferroni correction; *P*-values lower than 0.05 were considered significant. Abbreviations: gRNA: genomic RNA; sgRNA: subgenomic RNA; IHC: immune histochemistry; VT: infectious viral titration.

|  |  | **SPECIE** | | | **SEX** | | | **TREATMENT** | | |
| --- | --- | --- | --- | --- | --- | --- | --- | --- | --- | --- |
| **TECHNIQUES** | **RESULT** | **% Hamster** | **% Mouse** | **p-value** | **% Female** | **% Male** | **p-value** | **% Treated** | **% Non-treated** | ***P*-value** |
| **gRNA - sgRNA** | **Agree** | 75.2 | 61.3 | 0.2080 | 73.7 | 69.8 | 1.000 | 70.1 | 73.3 | 0.2080 |
|  | **Disagree** | 24.8 | 38.7 |  | 26.3 | 30.2 |  | 29.9 | 26.7 |  |
| **gRNA - VT** | **Agree** | 56.5 | 68.9 | 0.4167 | 58.1 | 60.4 | 1.000 | 57.2 | 60.1 | 0.4167 |
|  | **Disagree** | 43.5 | 31.1 |  | 41.9 | 39.6 |  | 42.8 | 39.9 |  |
| **gRNA - IHC** | **Agree** | 87.3 | 74.0 | 0.1049 | 81.1 | 86.7 | 1.000 | 82.8 | 84.4 | 0.1049 |
|  | **Disagree** | 12.7 | 26.0 |  | 18.9 | 13.3 |  | 17.2 | 15.6 |  |
| **sgRNA - VT** | **Agree** | 76.0 | 84.2 | 0.8634 | 78.6 | 77.4 | 1.000 | 83.6 | 74.5 | 0.8634 |
|  | **Disagree** | 24.0 | 15.8 |  | 21.4 | 22.6 |  | 16.4 | 25.5 |  |
| **sgRNA - IHC** | **Agree** | 81.1 | 70.5 | 0.4735 | 80.5 | 75.3 | 1.000 | 83.6 | 72.1 | 0.4735 |
|  | **Disagree** | 18.9 | 29.5 |  | 19.5 | 24.7 |  | 16.4 | 27.9 |  |
| **VT -IHC** | **Agree** | 78.8 | 79.7 | 1.000 | 81.8 | 75.5 | 1.000 | 79.5 | 78.5 | 1.000 |
|  | **Disagree** | 21.2 | 20.3 |  | 18.2 | 24.5 |  | 20.5 | 21.5 |  |

**Supplementary Table 2 (A to D). Analyses performed on the hamster dataset.** Abbreviations: gRNA: genomic RNA; sgRNA: subgenomic RNA; IHC: immune histochemistry; VT: infectious viral titration; CI: confidence interval; dpi: days post inoculation; L: lung: NT: nasal turbinates; OS: oropharyngeal swab; N/A: not applicable.

1. **Cohen’s k coefficients**

| Techniques | N | Proportion of agreement | Kappa | 95% CI |
| --- | --- | --- | --- | --- |
| **gRNA vs sgRNA** | 547 | 78.79% | 0.33 | [0.27, 0.39] |
| **gRNA vs VT** | 637 | 54.16% | 0.08 | [0.05, 0.12] |
| **gRNA vs IHC** | 274 | 86.50% | 0.39 | [0.28, 0.51] |
| **sgRNA vs VT** | 482 | 70.95% | 0.42 | [0.35, 0.50] |
| **sgRNA vs IHC** | 270 | 80.00% | 0.46 | [0.35, 0.57] |
| **VT vs IHC** | 246 | 76.42% | 0.38 | [0.28, 0.48] |

1. **No differences between sexes nor between treatment status**

|  |  | **SEX** | | | **TREATMENT** | | |
| --- | --- | --- | --- | --- | --- | --- | --- |
| **TECHNIQUES** | **RESULT** | **% Female** | **% Male** | ***P*-value** | **% Treated** | **% Non-treated** | ***P* -value** |
| **gRNA - sgRNA** | **Agree** | 78.3 | 75.3 | 1.000 | 69.0 | 81.8 | 1.000 |
|  | **Disagree** | 21.7 | 24.7 |  | 31.0 | 18.2 |  |
| **gRNA - VT** | **Agree** | 54.6 | 59.5 | 1.000 | 56.2 | 57.2 | 1.000 |
|  | **Disagree** | 45.4 | 40.5 |  | 43.8 | 42.8 |  |
| **gRNA - IHC** | **Agree** | 82.6 | 90.1 | 0.7290 | 82.7 | 89.3 | 1.000 |
|  | **Disagree** | 17.4 | 9.9 |  | 17.3 | 10.7 |  |
| **sgRNA - VT** | **Agree** | 74.1 | 76.8 | 1.000 | 85.1 | 70.0 | 1.000 |
|  | **Disagree** | 25.9 | 23.2 |  | 14.9 | 30.0 |  |
| **sgRNA - IHC** | **Agree** | 81.9 | 79.4 | 1.000 | 82.1 | 79.3 | 1.000 |
|  | **Disagree** | 18.1 | 20.6 |  | 17.9 | 20.7 |  |
| **VT -IHC** | **Agree** | 81.4 | 80.5 | 1.000 | 79.8 | 82.3 | 1.000 |
|  | **Disagree** | 18.6 | 19.5 |  | 20.2 | 17.7 |  |

1. **Differences between dpi**

| ***P*-values** | **2 dpi - 4 dpi** | **2 dpi – 6-7 dpi** | **4 dpi – 6-7 dpi** |
| --- | --- | --- | --- |
| **gRNA-sgRNA** | 0.9835 | 0.0000 | 0.0000 |
| **gRNA-VT** | 10.000 | 0.0000 | 0.0000 |
| **gRNA-IHC** | 10000 | 0.0000 | 0.0000 |
| **sgRNA-VT** | 10.000 | 0.7012 | 10000 |
| **sgRNA-IHC** | 10000 | 0.0000 | 0.0000 |
| **VT-IHC** | 10000 | 0.0000 | 0.0000 |

1. **Differences between sample types**

| **P-values** | **L - NT** | **L - OS** | **NT - OS** |
| --- | --- | --- | --- |
| **gRNA-sgRNA** | 1.000 | 1.000 | 1.000 |
| **gRNA-VT** | 1.000 | 0.0000 | 0.0000 |
| **gRNA-IHC** | 1.000 | N/A | N/A |
| **sgRNA-VT** | 0.0781 | 0.0000 | 0.0024 |
| **sgRNA-IHC** | 1.000 | N/A | N/A |
| **VT-IHC** | 1.000 | N/A | N/A |

**Supplementary Table 3 (A to E). Analyses performed on the mouse dataset.** Abbreviations: gRNA: genomic RNA; sgRNA: subgenomic RNA; IHC: immune histochemistry; VT: infectious viral titration; CI: confidence interval; dpi: days post inoculation; L: lung: NT: nasal turbinates; OS: oropharyngeal swab; N/A: not applicable.

**A) Cohen’s k coefficients**

| Techniques | N | Proportion of agreement | Kappa | 95% CI |
| --- | --- | --- | --- | --- |
| **gRNA vs sgRNA** | 547 | 78.79% | 0.33 | [0.27, 0.39] |
| **gRNA vs VT** | 637 | 54.16% | 0.08 | [0.05, 0.12] |
| **gRNA vs IHC** | 274 | 86.50% | 0.39 | [0.28, 0.51] |
| **sgRNA vs VT** | 482 | 70.95% | 0.42 | [0.35, 0.50] |
| **sgRNA vs IHC** | 270 | 80.00% | 0.46 | [0.35, 0.57] |
| **VT vs IHC** | 246 | 76.42% | 0.38 | [0.28, 0.48] |

**B) Differences between sexes**

|  |  | **SEX** | | |
| --- | --- | --- | --- | --- |
| **TECHNIQUES** | **RESULT** | **% Female** | **% Male** | ***P*-value** |
| **gRNA - sgRNA** | **Agree** | 65.0 | 55.4 | 0.9944 |
|  | **Disagree** | 35.0 | 44.6 |  |
| **gRNA – VT** | **Agree** | 70.3 | 66.7 | 1.000 |
|  | **Disagree** | 29.7 | 33.3 |  |
| **gRNA – IHC** | **Agree** | 76.6 | 69.6 | 1.000 |
|  | **Disagree** | 23.4 | 30.4 |  |
| **sgRNA – VT** | **Agree** | 89.0 | 76.4 | 0.1115 |
|  | **Disagree** | 11.0 | 23.6 |  |
| **sgRNA – IHC** | **Agree** | 77.6 | 58.7 | 0.0243 |
|  | **Disagree** | 22.4 | 41.3 |  |
| **VT -IHC** | **Agree** | 85.1 | 70.5 | 0.0750 |
|  | **Disagree** | 14.9 | 29.5 |  |

**C) No differences between treatment status**

|  |  | **TREATMENT** | | |
| --- | --- | --- | --- | --- |
| **TECHNIQUES** | **RESULT** | **% Treated** | **% Non-treated** | ***P*-value** |
| **gRNA - sgRNA** | **Agree** | 62.0 | 57.1 | 1.000 |
|  | **Disagree** | 38.0 | 42.9 |  |
| **gRNA - VT** | **Agree** | 69.8 | 64.3 | 1.000 |
|  | **Disagree** | 30.2 | 35.7 |  |
| **gRNA - IHC** | **Agree** | 72.5 | 81.0 | 1.000 |
|  | **Disagree** | 27.5 | 19.0 |  |
| **sgRNA - VT** | **Agree** | 84.0 | 85.7 | 1.000 |
|  | **Disagree** | 16.0 | 14.3 |  |
| **sgRNA - IHC** | **Agree** | 69.6 | 75.0 | 1.000 |
|  | **Disagree** | 30.4 | 25.0 |  |
| **VT -IHC** | **Agree** | 80.6 | 75.0 | 1.000 |
|  | **Disagree** | 19.4 | 25.0 |  |

**D) Differences between dpi**

| ***P*-values** | **2 dpi - 4 dpi** | **2 dpi – 6-7 dpi** | **4 dpi – 6-7 dpi** |
| --- | --- | --- | --- |
| **gRNA -sgRNA** | 1.000 | 0.3144 | 0.0059 |
| **gRNA -VT** | 10000 | 0.0943 | 0.9400 |
| **gRNA - IHC** | 0.5165 | 0.4205 | 1.000 |
| **sgRNA - VT** | 1.000 | 0.0478 | 0.4892 |
| **sgRNA - IHC** | 1.000 | 1.000 | 1.000 |
| **VT - IHC** | 0.6044 | 0.0061 | 0.5098 |

**E) Differences between sample types**

| ***P*-values** | **B - L** | **B - NT** | **B - OS** | **L - NT** | **L - OS** | **NT - OS** |
| --- | --- | --- | --- | --- | --- | --- |
| **gRNA -sgRNA** | 1.000 | 0.0466 | 0.0000 | 0.0023 | 0.0000 | 0.0000 |
| **gRNA - VT** | 1.000 | 0.0374 | 0.0000 | 0.9313 | 0.0000 | 0.0000 |
| **-gRNA – IHC** | 0.0933 | 0.0000 | N/A | 0.0038 | N/A | N/A |
| **sgRNA -VT** | 1.000 | 0.6558 | 0.0845 | 0.0382 | 0.8600 | 0.0007 |
| **sgRNA-IHC** | 0.0050 | 0.0000 | N/A | 1.000 | N/A | N/A |
| **VT-IHC** | 0.0000 | 0.0000 | N/A | 1.000 | N/A | N/A |

**Supplementary Figure 1 (A to D).** Abbreviations**:** gRNA: genomic RNA; sgRNA: subgenomic RNA; IHC: immunohistochemistry**;** F: females; M: males**;** dpi: days post-inoculation L: lung: NT: nasal turbinates.

1. **
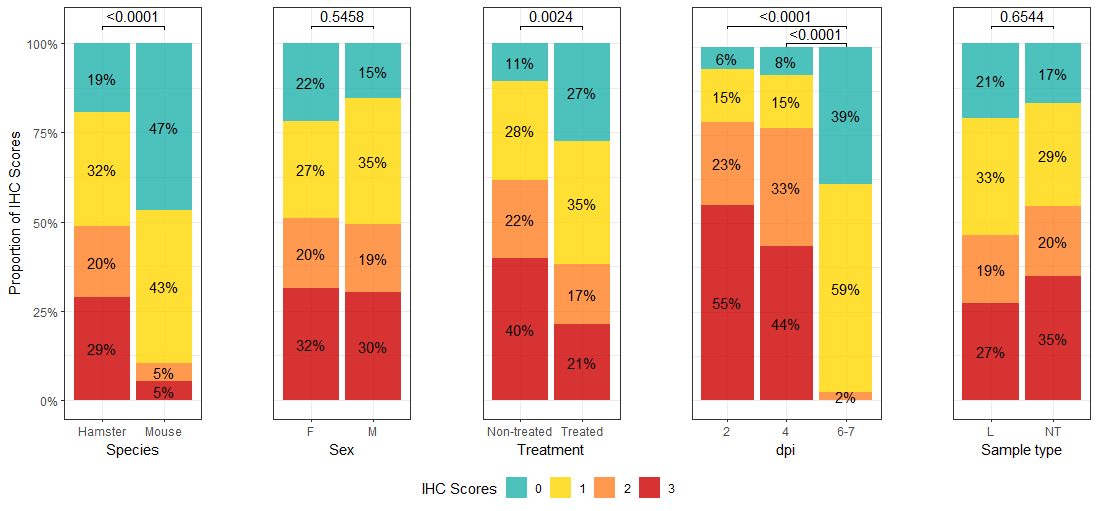
Differences in the proportion of IHC scores across treatment status and time points in the hamster dataset.** Chi-squared test followed by Bonferroni correction; *P-*values lower than 0.05 were considered significant.
2. **
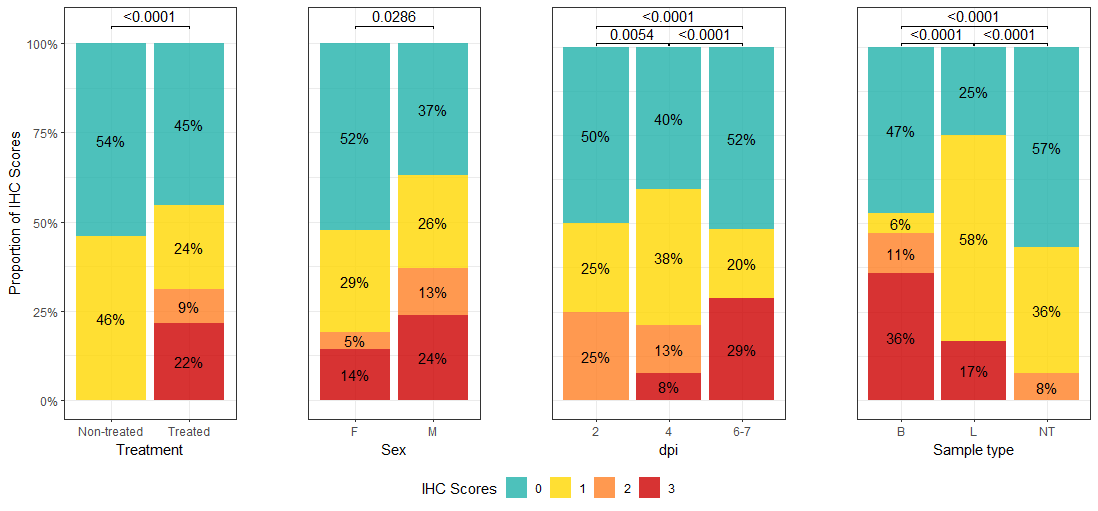
Differences in the proportion of IHC scores across treatment status, sexes, time points and sample types in the mice dataset.** Chi-squared test followed by Bonferroni correction; *P-*values lower than 0.05 were considered significant.
3. **Comparison of the distribution of (left to right) standardised gRNA, sgRNA and infectious viral titration results by IHC scores in the hamster dataset.** Kruskal-Wallis test and Dunn’s test for multiple comparisons with Bonferroni correction; *P*-values lower than 0.05 were considered significant.

**
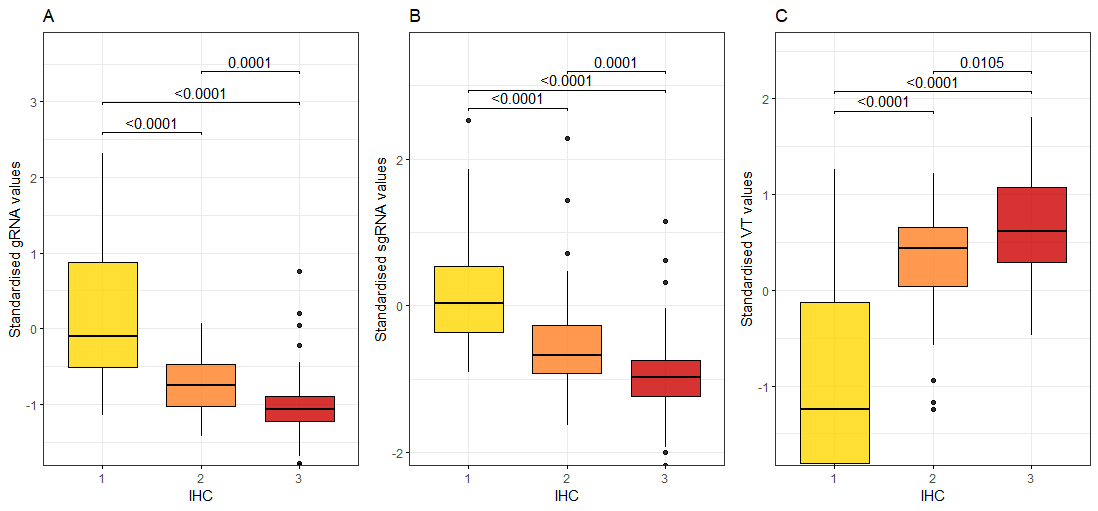
**

1. **Comparison of the distribution of (left to right) standardised gRNA, sgRNA and infectious viral titration results by IHC scores in the mice dataset.** Kruskal-Wallis test and Dunn’s test for multiple comparisons with Bonferroni correction; *P*-values lower than 0.05 were considered significant.


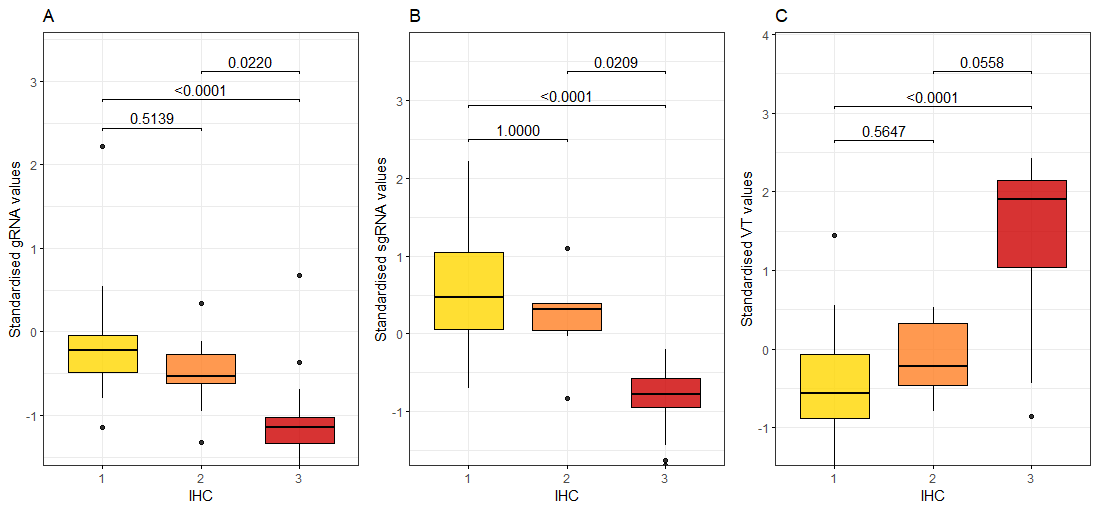


**Supplementary Figure 2. Influence of day post-infection on the percentage of agreement between techniques.** Chi-squared test followed by Bonferroni correction; P-values lower than 0.05 were considered significant. Abbreviations**:** gRNA: genomic RNA; sgRNA: subgenomic RNA; IHC: immunohistochemistry**;** VT: infectious viral titration; dpi: days post-inoculation.

**
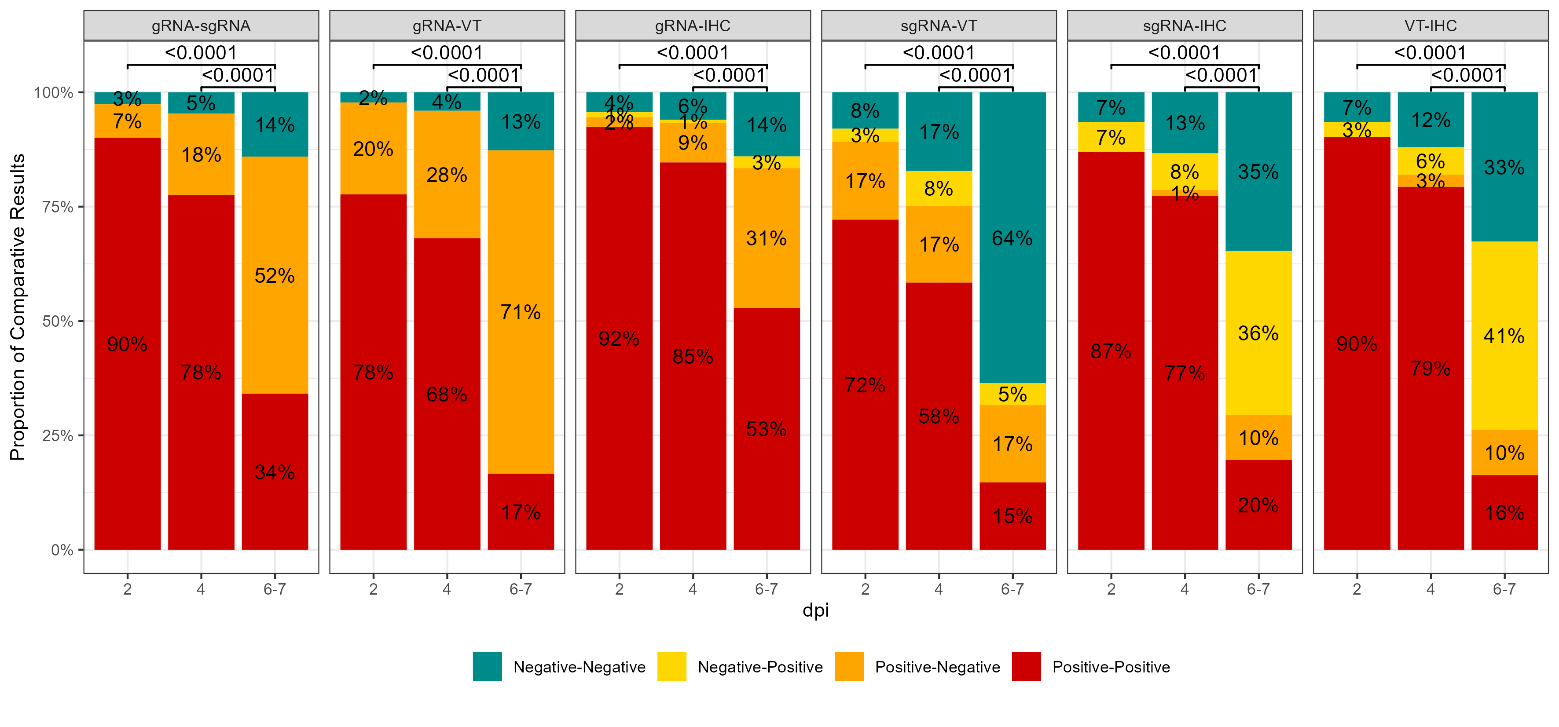
**
